# Acupuncture improved hepatic steatosis in HFD-induced NAFLD rats by regulating intestinal microbiota

**DOI:** 10.3389/fmicb.2023.1131092

**Published:** 2023-03-16

**Authors:** Haiying Wang, Qiang Wang, Cuimei Liang, Liang Pan, Hui Hu, Hongjuan Fang

**Affiliations:** ^1^Department of Cardiology, Dongzhimen Hospital, Beijing University of Chinese Medicine, Beijing, China; ^2^Chinese People’s Liberation Army Center of Disease Control and Prevention, Beijing, China; ^3^Department of Acupuncture, Dongfang Hospital, Beijing University of Chinese Medicine, Beijing, China; ^4^Department of Endocrinology, Beijing Tiantan Hospital, Capital Medical University, Beijing, China

**Keywords:** non-alcoholic fatty liver disease, intestinal microbiota, acupuncture, hepatic steatosis, inflammation

## Abstract

**Background:**

Intestinal dysbiosis has been increasingly implicated in the pathogenesis of non-alcoholic fatty liver disease (NAFLD). Acupuncture has been shown to have beneficial effects on NAFLD, but the mechanism is not yet clear. This study explores the potential beneficial effects of acupuncture on intestinal microbiota in NAFLD.

**Methods:**

An NAFLD model in Sprague Dawley rats was established using a high-fat diet (HFD) for 10  weeks. NAFLD rats were randomly divided into control, model, and acupuncture groups. Following acupuncture treatment over 6 weeks, automated biochemical analysis was used to measure serum lipid metabolism parameters, including levels of alanine transferase, aspartate transferase, alkaline phosphatase, total cholesterol, triglycerides, high-density lipoprotein cholesterol, and low-density lipoprotein cholesterol. The level of serum inflammatory factors interleukin (IL)-6, IL-10, and tumor necrosis factor-alpha (TNF-α) were measured by enzyme-linked immunosorbent assay. The characteristics of steatosis were evaluated using quantitative computed tomography, hematoxylin and eosin staining, and Oil Red O staining in the liver, while the intestinal microbiota was determined using 16S rRNA gene sequencing.

**Results:**

Acupuncture decreased the systemic inflammatory response, ameliorated dyslipidemia, and improved liver function indexes in NAFLD model rats. Tomography and staining indicated that acupuncture reduced steatosis and infiltration of inflammatory cells in the liver. 16S rRNA analysis showed that acupuncture reduced the Firmicutes to Bacteroidetes (F/B) ratio, increased the abundance of microbiota, including Bacteroidales_S24-7_group, Prevotellaceae, Bacteroidaceae, Blautia, norank_f_Bacteroidales_S24-7_group, Bacteroides, and Prevotella_9, and decreased the abundance of Ruminococcaceae_UCG-014. Correlation analysis suggested a close correlation between lipid metabolism, inflammation factors, hepatic steatosis, and the changed intestinal microbiota.

**Conclusion:**

Acupuncture can significantly improve lipid metabolism and the systemic inflammatory response in HFD-induced NAFLD rats, potentially by regulating intestinal microbiota composition.

## 1. Introduction

Non-alcoholic fatty liver disease (NAFLD) is recognized as a major public health threat, and its incidence continues to grow annually, reaching 25.2% worldwide (Asrani et al., 2019). As the top contributor to rapid increases in mortality due to liver-related diseases (Paik et al., 2020), NAFLD has become the primary indication for end-stage liver disease (Estes et al., 2018), primary hepatocellular carcinoma (Wong et al., 2014), and liver transplantation (Noureddin et al., 2018). In a longitudinal cohort study with a 14.2-year follow-up, researchers found that even the liver steatosis simplex at an early stage (triglyceride accumulated in the cytoplasm of non-fat cells) increased the mortality risk by 71%, and this risk is positively related to NAFLD severity (Simon et al., 2021). NAFLD is closely related to metabolic syndromes such as obesity, insulin resistance, hyperlipidemia, and hypertension (Ballestri et al., 2016). Epidemiology studies with data from 20 different countries confirmed that NAFLD prevalence is twice as high among those with type 2 diabetes compared with that of the general population (Younossi et al., 2019). Although many drug trials are currently evaluating therapies for NAFLD, none have been approved for clinical use. Hence, there is an urgent need to identify safe and efficient interventions.

As proven by increasing evidence, NAFLD occurrence and development are driven by gut–liver axis unbalance and metabolites of intestinal microbiota (Sharpton et al., 2019). A study found that when the feces of obese women with NAFLD were planted into the intestinal tract of rats fed with a general diet, the liver triglyceride content of the rats increased in 14 days (Hoyles et al., 2018). Le Roy et al. reported that when germ-free mice underwent implantation of feces from hyperglycemic mice and were fed a high-fat diet (HFD) for 16 weeks, bullous steatosis was found in their liver cells (Le Roy et al., 2013). This indicates the critical role played by intestinal microbiota in NAFLD occurrence and development. Furthermore, when NAFLD mice induced by an HFD were treated with probiotics such as *Lactobacillus lactis* and *Pediococcus* for 8 weeks, NAFLD symptoms improved (Yu et al., 2021). Given sizable animal models and human studies demonstrating the role of intestinal microbiota in NAFLD development, it is important to determine whether specific bacterial strains or community composition drives disease progression.

As an alternative therapy with a history spanning several thousand years, acupuncture plays a key role in traditional Chinese medicine. Several studies confirmed that acupuncture could effectively alleviate hepatic steatosis and improve glucolipid metabolism and insulin resistance (Dong et al., 2020; Han et al., 2020). Acupuncturing various acupoints on HFD-induced NAFLD rats was found to effectively suppress the response of inflammatory factors such as tumor necrosis factor-alpha (TNF-α) and interleukin (IL)-6 (Yu et al., 2017), thus suppressing inflammation. Acupuncture intervention by Zhang et al. on HFD-induced obesity mouse models effectively alleviated oxidative stress and liver cell apoptosis (Zhang et al., 2020), effectively inhibiting NAFLD progression. Various studies confirmed that acupuncture therapy is valid and safe for alleviating NAFLD (Chen et al., 2021), yet there are few reports on whether acupuncture can improve NAFLD by regulating intestinal microbiota. To better understand the mechanism of acupuncture therapy for NAFLD, we established a NAFLD rat model by HFD feeding. Observations of intestinal microbiota changes in NAFLD rats following acupuncture treatment could support research on the potential mechanism of acupuncture in managing NAFLD.

## 2. Materials and methods

### 2.1. Experimental animals

We purchased 30 healthy male Sprague–Dawley (SD) rats (age: 5–6 weeks old, body mass: 220 ± 20 g) from the Experimental Animal Centre of the Chinese People’s Liberation Army Academy of Military Medical Sciences, Beijing, China. All rats were allowed to feed and drink freely, with feed and water replaced once every other day. A standard specific pathogen-free environment was established with 22 ± 2°C ambient temperature, 50–60% relative humidity, and 12 h of alternating lighting and darkness. The Animal Experiments and Experimental Animal Welfare Committee of the Chinese People’s Liberation Army Center of Disease Control and Prevention approved the experimental protocol, and the study process followed all ethical review guidelines.

### 2.2. Establishment of NAFLD rat model

The rats were randomly divided into control (*n* = 11) and model (*n* = 19) groups after a week of adjustable feeding. The control group was fed with a normal chow diet (NCD: 24% corn flour, 20% bran, 20% bean cake, 20% four, 6% cellulose, 5% fish meal, 3% bone meal, and 2% salt; Experiment Animal Center of Military Medical Science Academy), while the model group was fed with an HFD (2.5% cholesterol, 0.3% sodium cholate, 20% saccharose, 20% lard, and 57.2% basal feed; Hua Fukang Biotechnology Co., Ltd., Beijing, China). Thereafter, three rats each were randomly selected from the control and model groups for a liver and spleen quantitative computed tomography (QCT) scan. Hematoxylin and eosin (H&E) and Oil Red O staining were performed on the liver tissue to measure liver steatosis and confirm the NAFLD rat model.

### 2.3. Acupuncture intervention

**Figure 1:**
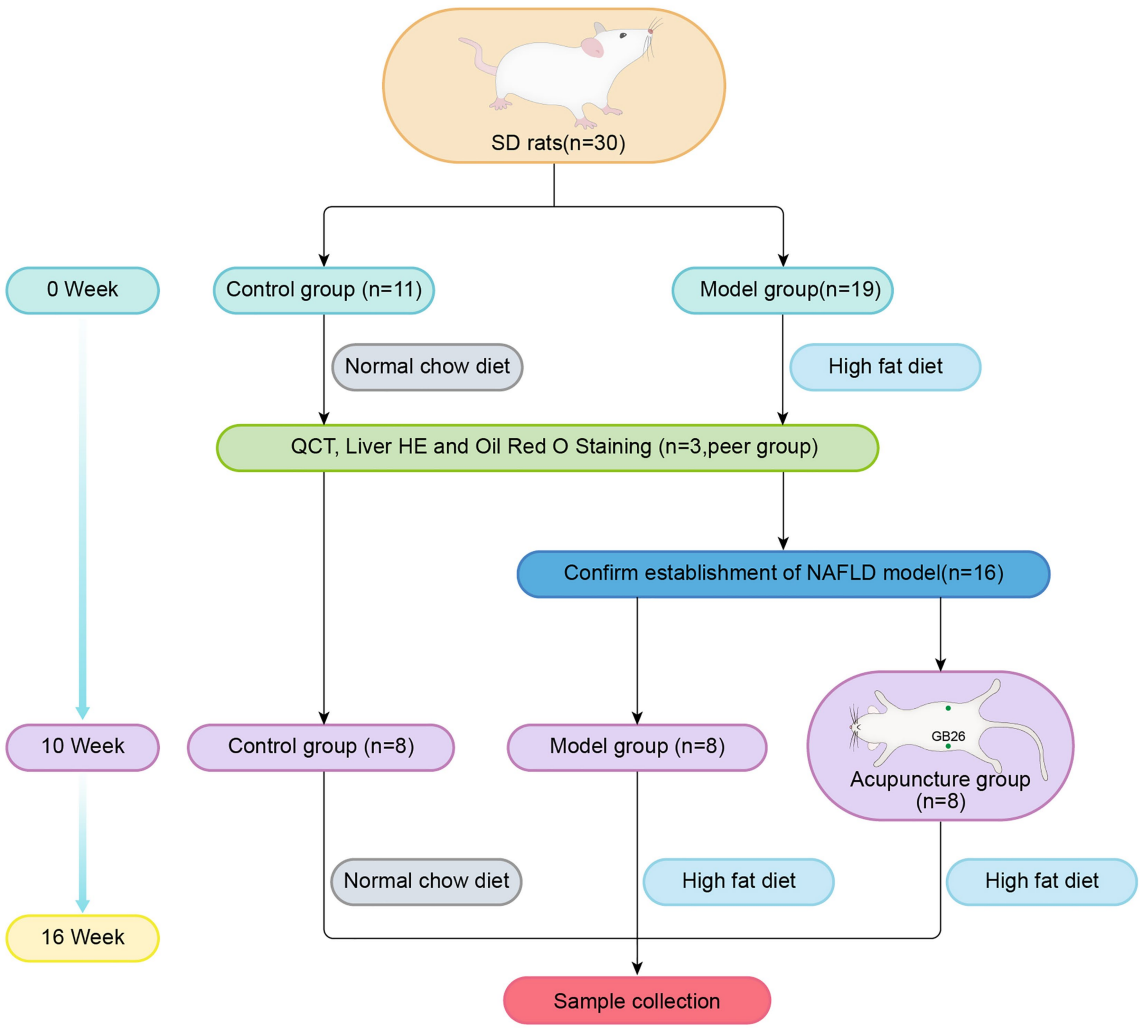
Groups and experimental procedure.

We randomly divided 16 NAFLD rats into the model (*n* = 8) and acupuncture (*n* = 8) groups. Both groups were fed the HFD, while the control group (*n* = 8) was fed the NCD. The experimental procedure is illustrated in Figure 1.

Acupoints with the bilateral “Daimai” (GB 26) were selected. The rats of the acupuncture group were placed and fixed in self-made conical rat bags. Sterile disposable acupuncture needles (Zhongyan Taihe Medicine Company, Ltd., Beijing, China; 0.30 mm × 25 mm) were inserted vertically into the bilateral GB 26 acupoints at a depth of 4–5 mm. The GB 26 acupoints were connected with the positive and negative poles of an electric acupuncture apparatus (Yingdi Electronic Medical Appliances Co., Ltd., Changzhou, China), with the following parameters: dilatational wave, frequency 2 Hz/15 Hz, and strength 1.5 mA. Treatments were performed on alternate weekdays (Monday/Wednesday/Friday) over 6 weeks. Each treatment lasted 20 min. During acupuncture, rats of the model group were fixed at the same time for 20 min without treatment, and the rats in the control group received no intervention.

### 2.4. Serum biochemical marker assay

All rats were anesthetized by intraperitoneal injection with 10% chloral hydrate (Sinopharm Chemical Reagent Co., Ltd., Shanghai, China) after fasting for 12 h. Blood samples were collected from the abdominal aorta and centrifuged at 4°C for 10 min at 3000 rpm. Next, the serum samples were refrigerated at −80°C until further analysis. The levels of serum lipid parameters, including total cholesterol (TC), triglycerides (TG), high-density lipoprotein cholesterol (HDL), and low-density lipoprotein cholesterol (LDL), as well as serum liver function parameters of alanine transferase (ALT), aspartate transferase (AST), and alkaline phosphatase (ALP) were measured using the MEK7222K Automated Hematology Analyzer (Nihon Kohden, Tokyo, Japan). Specific enzyme-linked immunosorbent assay kits were used to measure the levels of serum inflammatory factors, including IL-6, TNF-α, and IL-10, as per the manufacturer’s instructions (R&D Systems, Minneapolis, MN, USA).

### 2.5. QCT scan

Five rats were randomly selected from each group, and after anesthesia, the limbs of each rat were fixed horizontally on a plate. QCT was performed using a Toshiba Aquilion 80-slice CT Scanner (Toshiba, Tokyo, Japan) with a Mindways calibration phantom (Mindways Software, Austin, TX, USA). The scan covered the diaphragmatic dome to the symphysis pubis, avoiding the hepatic portal blood vessels and intra-abdominal fat tissues and keeping a distance from the liver edges. Liver fat content was determined as follows: the right posterior lobe of the hepatic portal level, regions of interest (ROI) 40 ± 0.5 mm^2^, spleen depth divided equally into three shares, measuring the middle-level center ROI 10 ± 0.2 mm^2^. Scan parameters: 0.985 pitch, 120 cm bed height, 120 kV, 250 mA, 1.0-mm thickness, 500 mm^2^ field of view, and standard reconstruction. The original images were uploaded to the CT workstation and analyzed using Mindways software version 4.2.

The QCT attenuation values were measured using the Hounsfeld Unit (HU) for both the liver and spleen, and hepatic steatosis was negatively correlated to liver attenuation values (Lee and Park, 2014). Liver CT (CT_L_) attenuation values were used to measure the liver fat content. Since the CT attenuation value of the spleen remained relatively stable, the ratio of the CT attenuation values of liver to spleen (CT_L/S_) was measured to quantitatively evaluate the severity of hepatic steatosis, being mild 0.7–1.0, moderate 0.5–0.7, and severe ≤0.5 (Zeng et al., 2008). All QCT operations were carried out by a radiologist recruited separately and blinded to the sample characteristics.

### 2.6. Histopathological liver assessment

#### 2.6.1. H&E staining

Following blood sampling, all the overnight fasted rats were sacrificed after being anesthetized, and whole fresh liver tissue was obtained and weighed for every rat. The liver samples were then fixed with 10% formaldehyde, embedded with paraffin, and cut into 2-μm thick sections. Some sections underwent H&E staining using standard techniques to observe morphological changes under a light microscope (IX81; Olympus, Tokyo, Japan).

#### 2.6.2. Oil Red O staining

The sections were subjected to Oil Red O according to the manufacturer’s instructions (Sinopharm Chemical Reagent Co., Ltd., Shanghai, China), and the distribution of liver lipid droplets was observed under a microscope (IX81; Olympus, Tokyo, Japan). Olympus Image-Pro Plus 6.0 was used to quantitatively analyze the stained regions.

### 2.7. Fecal 16S rRNA gene sequencing

Before rats were sacrificed, fecal samples were collected. Microbial community genomic DNA was extracted from fecal samples using an E.Z.N.A.® soil DNA Kit (Omega Bio-tek, Norcross, GA, USA) according to the manufacturer’s instructions. The DNA extract was checked using 1% agarose gel electrophoresis. The final concentration and purity of microbial DNA were measured using a NanoDrop 2000 UV–vis spectrophotometer (Thermo Scientific, Wilmington, NC, USA). The hypervariable region V3–V4 of the bacterial 16S rRNA gene was amplified using primer pairs 338F (5′-ACTCCT ACGGGAGGCAGCAG-3′) and 806R (5′-GGACTACHVGGGT WTCTAAT-3′) using an ABI GeneAmp® 9,700 PCR thermocycler (Applied Biosystems, Foster City, CA, USA). PCR amplification conditions were as follows: denaturation at 95°C for 3 min, 27 cycles of denaturation at 95°C for 30 s, annealing at 55°C for 30 s, extension at 72°C for 45 s, single extension at 72°C for 10 min, and a final extension at 72°C for 10 min. An AxyPrep DNA Gel Extraction Kit (Axygen Biosciences, Union City, CA, USA) was used for PCR purification. A Quantus™ Fluorometer (Promega, Madison, WI, USA) was used for quantitative testing. Purified amplicons were pooled in equimolar concentrations, and paired-end sequencing was carried out using an Illumina MiSeq PE 300 platform (Illumina, San Diego, CA, USA).

### 2.8. Microbial analysis of fecal 16S rRNA gene sequencing

The raw sequencing reads were demultiplexed, filtered, and merged using FLASH software version 1.2.7 (Magoč and Salzberg, 2011). Operational taxonomic units (OTUs) with 97% similarity cutoff were assigned to the same OTUs using Uparse, and chimeric sequences were identified and deleted using the UCHIME algorithm. Representative sequences for each OTU were analyzed using RDP Classifier at a confidence threshold of 0.7 with the 16S rRNA database (Silva v128). Relative OTU abundances were normalized using a standard sequence number corresponding to the sample with the least sequences and then used for diversity analysis. Rank-abundance curves were used to assess species richness and evenness. Alpha diversity analysis was conducted using Shannon and Sobs indices, and the rarefaction curve of the Shannon index was evaluated. Community composition was visualized as bar plots for phylum, family, and genus levels. Partial least squares discriminant analysis (PLS-DA) was conducted to determine species similarity and distinction. Distance-based redundancy analysis (db-RDA) was used to evaluate the correlation between physiological data (lipid metabolism parameters, inflammatory factors, and hepatic steatosis indicators) and gut microbiota. Spearman correlation was illustrated *via* heatmap.

### 2.9. Statistical analysis

SPSS 20.0 (SPSS Inc., Chicago, IL, USA) was used to analyze the data, and the statistical description of variables is shown as the mean ± standard deviation. Normally distributed data were tested by one-way analysis of variance (ANOVA) followed by least significant difference (LSD) analysis; non-normally distributed data were analyzed using the nonparametric Kruskal–Wallis test. Changes in the composition of intestinal microflora were evaluated using the Kruskal–Wallis *H*-test, Wilcoxon rank-sum test, or Mann–Whitney *U*-test. Correlation analysis was performed using Spearman correlation. *p* < 0.05 was considered statistically significant.

## 3. Results

### 3.1. Acupuncture improved metabolic disorders and inflammatory response in NAFLD rats

**Figure 2:**
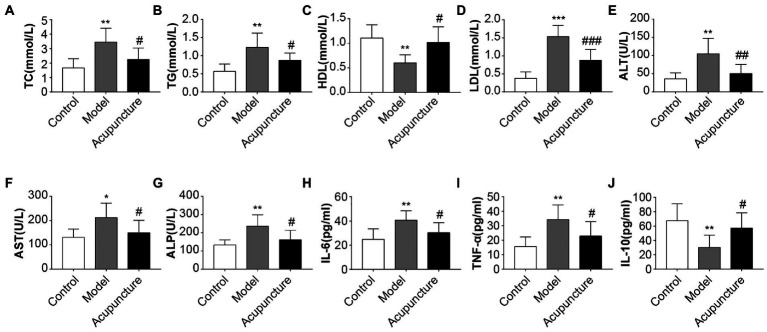
Impact of acupuncture on serum lipid metabolism and inflammation in non-alcoholic fatty liver disease (NAFLD) rats. Serum lipid metabolism parameters **(A–G)**, Serum inflammatory factors **(H–J)**. **(A)** Serum total cholesterol (TC). **(B)** Serum triglycerides (TG). **(C)** Serum high-density lipoprotein cholesterol (HDL). **(D)** Serum low-density lipoprotein cholesterol (LDL). **(E)** Serum alanine transferase (ALT). **(F)** Serum aspartate transferase (AST). **(G)** Serum alkaline phosphatase (ALP). **(H)** Serum interleukin (IL)-6. **(I)** Serum tumor necrosis factor-alpha (TNF-α). **(J)** Serum interleukin (IL)-10. Data are shown as mean ± SD. ^*^p < 0.05, ^**^p < 0.01, ^***^p < 0.001, Control vs. Model; ^#^*p* < 0.05, ^##^*p* < 0.01, ^###^*p* < 0.001, Model vs. Acupuncture.

Compared with the control group, the model group exhibited significant increases in serum TC, TG (both *p* < 0.01, Figures 2A,B), LDL (*p* < 0.001, Figure 2D), ALT, ALP (both *p* < 0.01, Figures 2E,G), AST (*p* < 0.05, Figure 2F) levels and a significant decrease in HDL levels (*p* < 0.01, Figure 2C). The acupuncture group revealed significant decreases in serum TC, TG, AST, and ALP (all *p* < 0.05, Figures 2A,B,F,G), LDL (*p* < 0.001, Figure 2D), and ALT (*p* < 0.01, Figure 2E) levels and a significant increase in HDL level (*p* < 0.05, Figure 2C) relative to the model group. The results suggest that acupuncture improved metabolic disorders in HFD-induced NAFLD rats.

The model group had significantly higher serum IL-6 and TNF-α levels (both *p* < 0.01, Figures 2H,I) and significantly lower IL-10 levels (*p* < 0.01, Figure 2J) than those of the control group. The acupuncture group showed significant decreases in IL-6 and TNF-α levels (both *p* < 0.05, Figures 2H,I) and a significant increase in IL-10 levels (*p* < 0.05, Figure 2J) compared with those of the model group. These results demonstrate that acupuncture suppresses the expression of proinflammatory factors IL-6 and TNF-α and increases that of the protective inflammatory factor IL-10.

### 3.2. Acupuncture ameliorated hepatic steatosis in NAFLD rats

**Figure 3:**
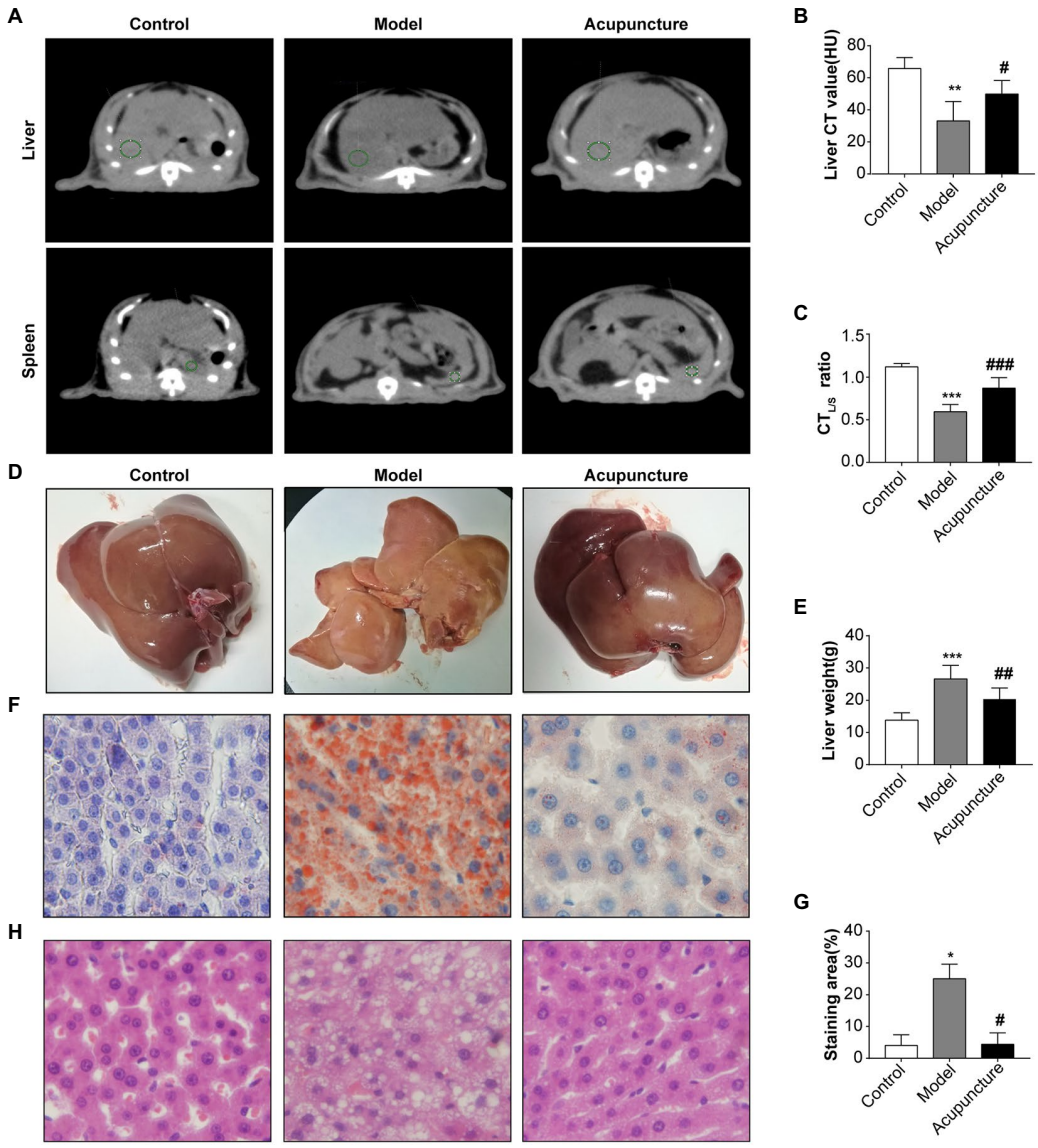
Impact of acupuncture on hepatic steatosis in non-alcoholic fatty liver disease (NAFLD) rats. **(A)** Liver and spleen CT value measurements of liver fat content. QCT films of the liver and spleen of the control, model, and acupuncture groups; green circles indicate regions of interest (ROI) in liver and spleen measurements used to indicate the CT values of the liver and spleen. **(B)** Liver CT (CT_L_) values (HU). **(C)** Liver to spleen CT ratio (CT_L/S_). **(D)** Fresh liver tissues. **(E)** Liver weight (g). **(F)** Oil Red O staining of rat livers (×100). **(G)** Quantification of the red area/total area by Oil Red O staining. **(H)** H&E staining of liver tissue (×100). Data are shown as mean ± SD. ^*^p < 0.05, ^***^p < 0.001, Control vs. Model, ^#^*p* < 0.05, ^##^*p* < 0.01, Model vs. Acupuncture.

CT_L_ attenuation values were analyzed using QCT (Figure 3A), yielding CT_L_ values of the control and model groups of 54.9 and 26.6 HU (<40), respectively, indicating a decrease in the model group compared with that of the control group (*p* < 0.01, Figure 3B). The CT_L_ value of the acupuncture group was 51.5 HU, indicating an increase compared with that of the model group (*p* < 0.05, Figure 3B). As shown in Figure 3C, in the control group, no rats exhibited hepatic steatosis (CT_L/S_ > 1); in the model group, three rats exhibited moderate liver steatosis (CT_L/S_: 0.5–0.7), and two rats exhibited severe hepatic steatosis (CT_L/S_: ≤ 0.5). In the acupuncture group, four rats exhibited mild hepatic steatosis (CT_L/S_: 0.7–1), and one rat exhibited moderate hepatic steatosis (CT_L/S_: 0.5–0.7). The CT_L/S_ of the model group was significantly decreased compared with that of the control group (*p* < 0.001, Figure 3C), while the CT_L/S_ of the acupuncture group was significantly increased compared with that of the model group (*p* < 0.001, Figure 3C). These results demonstrate that acupuncture significantly alleviates liver fat content, thus slowing the progress of hepatic steatosis.

Figure 3D shows fresh liver tissues. The liver color of the control group appeared dark red without obvious lipid particle accumulation. However, the liver of the model group appeared enlarged with an obvious yellowish-white color and visible lipid particle accumulation. The liver appearance and color of the acupuncture group were closer to those of the control group, with a visible reduction in lipid particles compared with the model group (Figure 3D). The liver weight of the model group was significantly increased compared with that of the control group (*p* < 0.001, Figure 3E), while that of the acupuncture group was significantly decreased relative to that of the model group (*p* < 0.01, Figure 3E).

Oil Red O staining confirmed that the hepatocyte nuclei in the control group appeared blue without obvious lipid deposition, while diffuse red lipid droplets within adjacent cells merged, and the lipid droplets compressed the nuclei in the model group (Figure 3F). Statistical analysis confirmed significantly increased lipid deposition in the model group (*p* < 0.05, Figure 3G) and significantly decreased deposition in the acupuncture group (*p* < 0.05, Figure 3G). The results of H&E staining are shown in Figure 3H. The control group did not display discernible hepatic steatosis, hepatocyte ballooning, lobular inflammation, or liver lobule structure disorder; however, the model group exhibited clear hepatic steatosis, hepatocyte ballooning, lobular inflammation, and liver lobule structure disorder. Compared with the model group, the acupuncture group demonstrated visible improvement in hepatic steatosis, hepatocyte ballooning, lobular inflammation, and liver lobule structure disorder.

### 3.3. Effects of acupuncture on community abundance and diversity of intestinal microbiota in NAFLD rats

16S rRNA sequencing was used to analyze intestinal microbiota in NAFLD rats. In total, 1,139,259 optimized sequences were obtained from 24 samples. The number of optimized-sequence bases (bp) was 469,868,757, the average number of sequences per sample was 47,469 bp, and the average sequence length was 412 bp (min_length: 216, max_length: 518).

**Figure 4:**
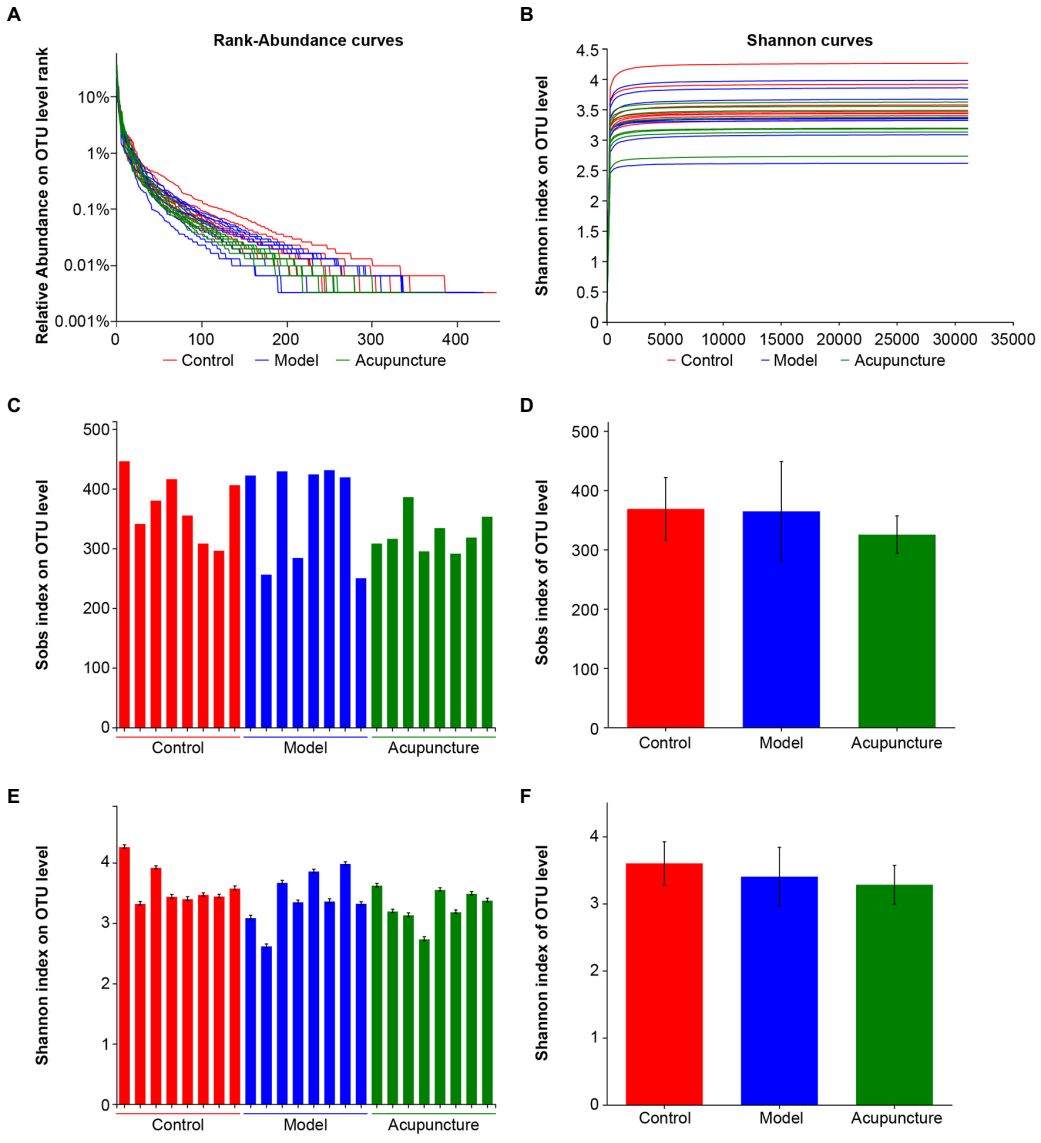
Sample sequencing results and species diversity analysis. **(A)** Rank-abundance curves indicating species diversity, including richness and evenness. Species richness is represented by the width of the curve. The larger the curve range on the horizontal axis, the higher the species richness; the smoother the curve, the more community evenness. **(B)** Rarefaction curve of Shannon index at the OTU level. **(C)** Alpha diversity of Sobs index at the OTU level indicating community richness. **(D)** Statistical analysis of the Sobs index at the OTU level. **(E)** Alpha diversity of the Shannon index at the OTU level indicating community diversity. **(F)** Statistical analysis of the Shannon index at the OTU level.

Rank-abundance curves were used to determine species diversity, including species richness and community evenness. The width and smoothness of curves obtained for the control, model, and acupuncture groups were similar. The results indicated no significant changes in species diversity among the three groups (Figure 4A). The rarefaction curve of the Shannon index was flat, indicating that the sequenced data were sufficient and community diversity was high (Figure 4B). Alpha diversity of Sobs and Shannon indices on the OTU level was also analyzed to determine community richness and diversity, respectively. Intestinal microbiota showed no statistically significant differences in community richness (Figures 4C,D) or diversity (Figures 4E,F) among the control, model, and acupuncture groups. These results indicate that neither the HFD diet nor acupuncture induce significant changes in microbiota community richness or diversity in NAFLD rats.

### 3.4. Effect of acupuncture on intestinal microbiota composition in NAFLD rats

**Figure 5:**
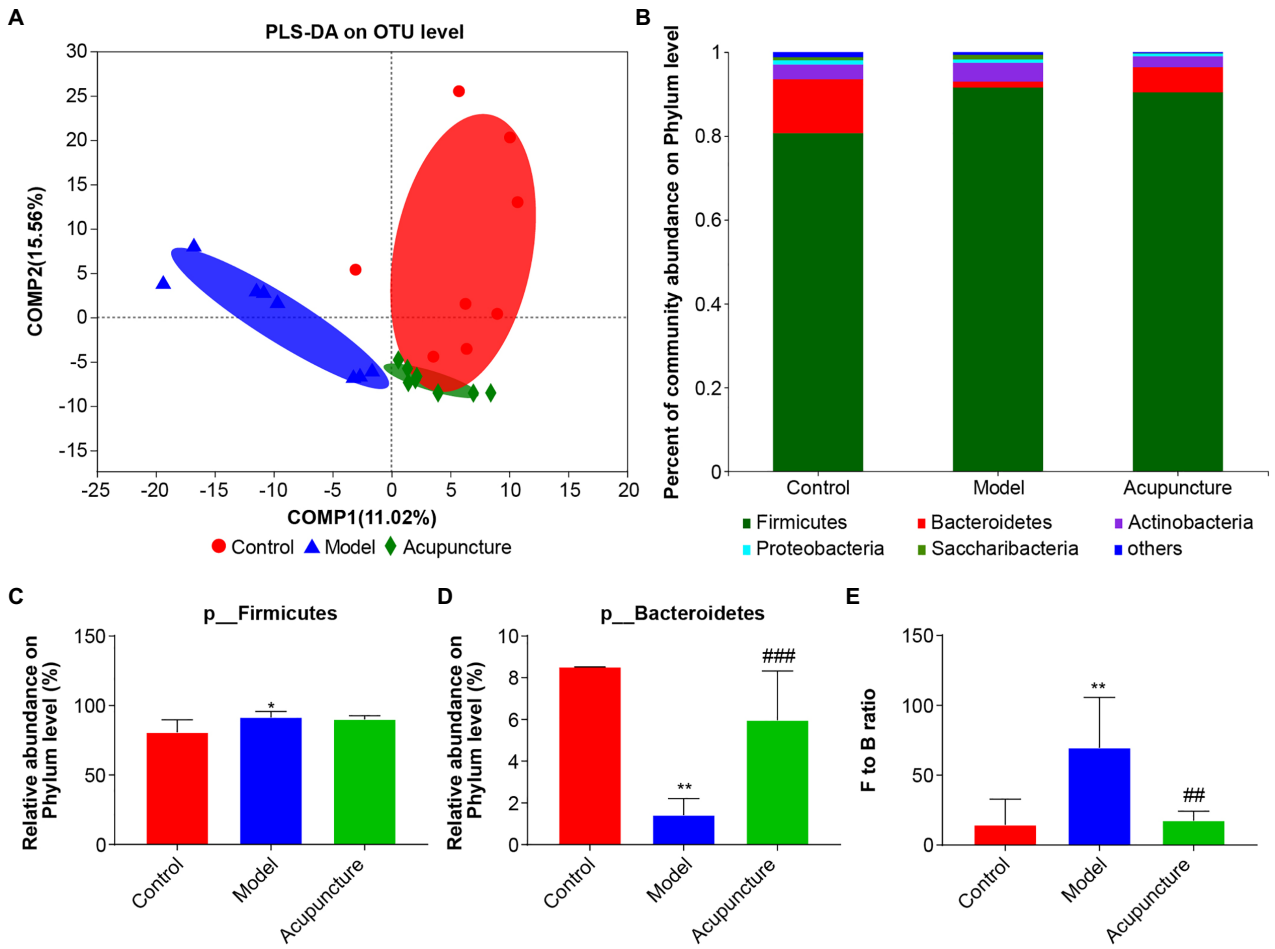
Regulating composition of the intestinal microbiota of non-alcoholic fatty liver disease (NAFLD) rats. **(A)** Partial least squares discriminant analysis (PLS-DA) at the OTU level. **(B)** Community bar plot analysis at the phylum level. “Others” represents a combination of all species with less than 0.01 abundance among all the samples. **(C)** Statistical difference analysis of Firmicutes among the control, model, and acupuncture groups. **(D)** Statistical difference analysis of Bacteroidetes among the three groups. **(E)** Statistical difference analysis of Firmicutes to Bacteroidetes (F/B) ratio. Data are shown as mean ± SD (*n* = 8 per group). ^*^p < 0.05, ^**^p < 0.01, Control vs. Model; ^##^*p* < 0.01, ^###^*p* < 0.001, Model vs. Acupuncture.

PLS-DA revealed that intestinal microbiota characteristics were distinct across control, model, and acupuncture groups at the OTU level (Figure 5A). The distribution of control group intestinal microbiota was closer to that of the acupuncture group, while both were distinct from that of the model group. To determine specific differences among the three groups, a bar plot was used to show the relative abundance of intestinal microbiota at phylum, family, and genus levels. The dominant microbiota phyla were Firmicutes and Bacteroidetes, with Actinobacteria, Proteobacteria, and Saccharibacteria also being abundant (Figure 5B). Statistical analysis showed that the relative abundance of Firmicutes in the model group was significantly higher than that of the control group (*p* < 0.05, Figure 5C), and the abundance of Firmicutes was not affected by acupuncture (Figure 5C). The relative abundance of Bacteroidetes in the model group was significantly decreased compared with that in the control group (*p* < 0.01, Figure 5D). The relative abundance of Bacteroidetes in the acupuncture group was significantly increased compared with that in the model group (*p* < 0.001, Figure 5D). The Firmicutes to Bacteroidetes (F/B) ratio of the model group was significantly higher compared with that of the control group (*p* < 0.01, Figure 5E), while the F/B ratio of the acupuncture group was significantly lower than that of the model group (*p* < 0.01, Figure 5E).

**Figure 6:**
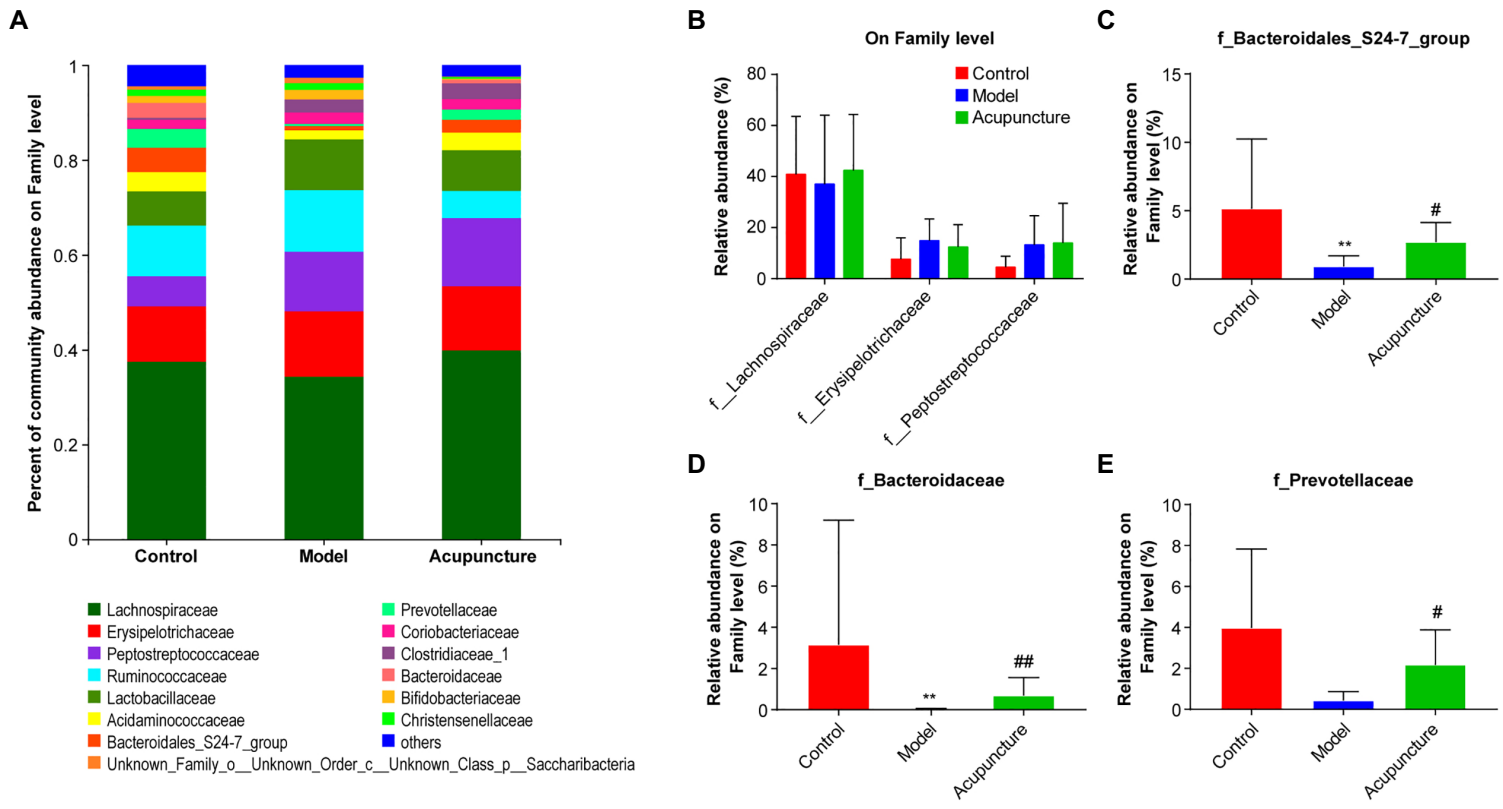
Impact of acupuncture on the composition of intestinal microbiota at the family level. **(A)** Community bar plot analysis. **(B)** Statistical difference analysis of the top three dominant microbiota families (Lachnospiraceae, Erysipelotrichaceae, and Peptostreptococcaceae) among all groups. **(C)** Bacteroidales_S24-7_group. **(D)** Bacteroidaceae. **(E)** Prevotellaceae. Data are shown as mean ± SD (*n* = 8 per group). ^**^p < 0.01, Control vs. Model; ^#^*p* < 0.05, ^##^*p* < 0.01, Model vs. Acupuncture.

The three most dominant microbiota families were Lachnospiraceae, Erysipelotrichaceae, and Peptostreptococcaceae, and no significant differences were observed among the three groups (Figures 6A,B). The relative abundances of Bacteroidales_S24-7_group and Bacteroidaceae in the model group were significantly decreased compared with those in the control group (both *p* < 0.01, Figures 6C,D). The relative abundances of Bacteroidales_S24-7_group (*p* < 0.05, Figure 6C) and Bacteroidaceae (*p* < 0.01, Figure 6D) in the acupuncture group were significantly increased compared with those in the model group. Compared with the control group, the relative abundance of Prevotellaceae was decreased in the model group, but this difference was not statistically significant (Figure 6E). Compared with the model group, the relative abundance of Prevotellaceae in the acupuncture group significantly increased (*p* < 0.05, Figure 6E).

**Figure 7:**
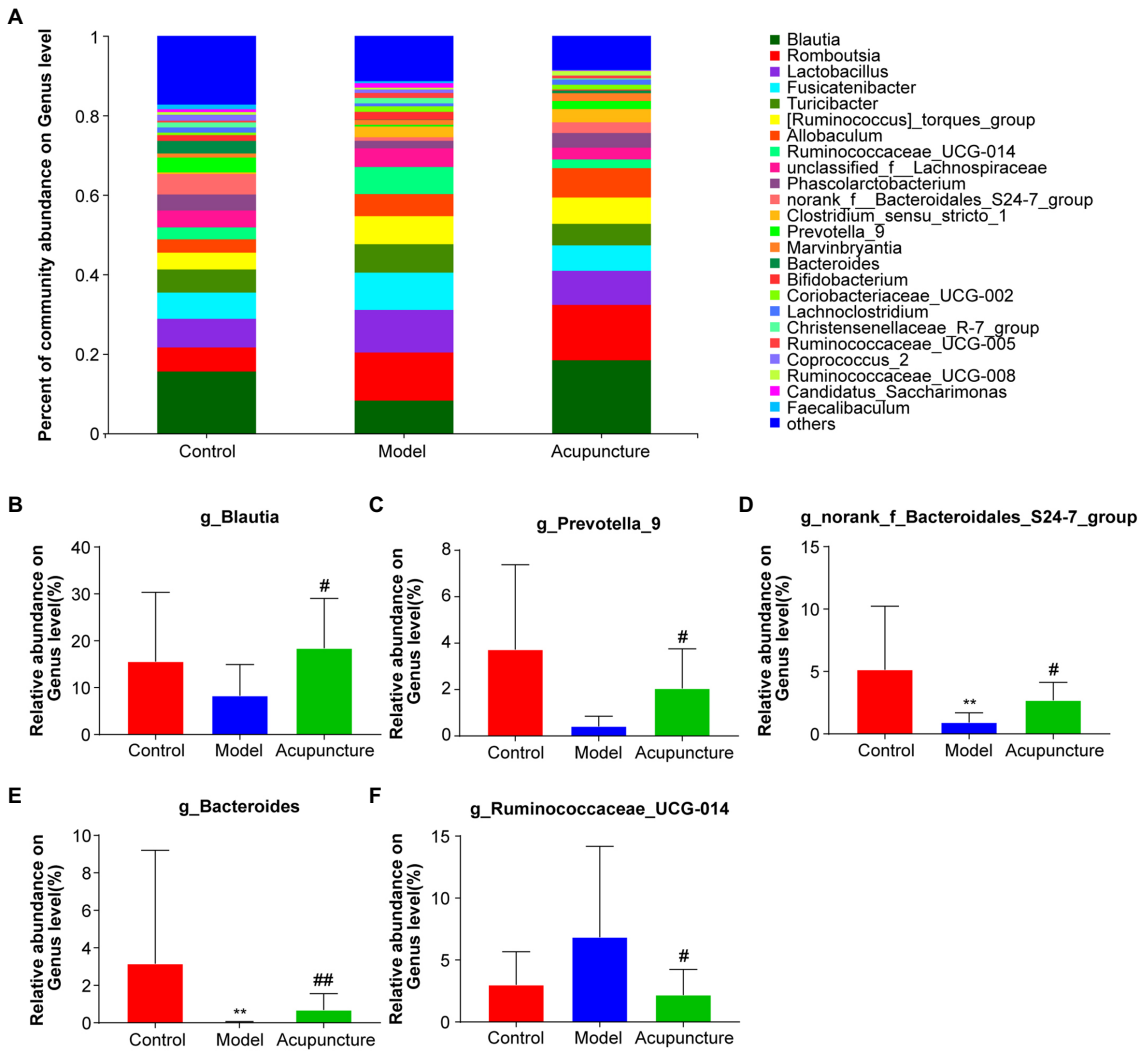
Modulation of intestinal microbiota composition by acupuncture at the genus level. **(A)** Community bar plot analysis of intestinal microbiota. **(B)** Blautia; **(C)** Prevotella_9. **(D)** norank_f_Bacteroidales_S24-7_group. **(E)** Bacteroides. **(F)** Ruminococcaceae_UCG-014. Data are shown as mean ± SD (*n* = 8 per group). ^**^p < 0.01, Control vs. Model, ^#^*p* < 0.05, ^##^*p* < 0.01, Model vs. Acupuncture.

At the genus level, a deeper specific intestinal microbiota composition was analyzed, additional types of genera were detected and the bar plot displayed the top 25 genera (Figure 7A), in which five main genera exhibited significant changes (Figures 7B–F). The relative abundances of Blautia and Prevotella_9 in the model group decreased compared with those in the control group, but this difference was not statistically significant (Figures 7B,C). However, the relative abundances of Blautia and Prevotella_9 (both *P* < 0.05, Figures 7B,C) in the acupuncture group significantly increased compared with those in the model group. The relative abundances of norank_f_Bacteroidales_S24-7_group and Bacteroides (both *P* < 0.01, Figures 7D,E) significantly decreased in the model group compared with those in the control group. The relative abundances of norank_f_Bacteroidales_S24-7_group (*P* < 0.05, Figure 7D) and Bacteroides (*P* < 0.01, Figure 7E) in the acupuncture group significantly increased compared with those in the model group. Compared with the control group, the relative abundance of Ruminococcaceae_UCG-014 increased in the model group, though this increase was not statistically significant (Figure 7F). The relative abundance of Ruminococcaceae_UCG-014 significantly decreased in the acupuncture group compared with that in the model group (*P* < 0.05, Figure 7F). Thus, acupuncture regulates intestinal microbiota composition at the phylum, family, and genus levels.

### 3.5. Correlation analysis of lipid metabolism parameters, inflammatory factors, and hepatic steatosis considering changes to intestinal microbiota

#### 3.5.1. Db-RDA at the genus level

**Figure 8:**
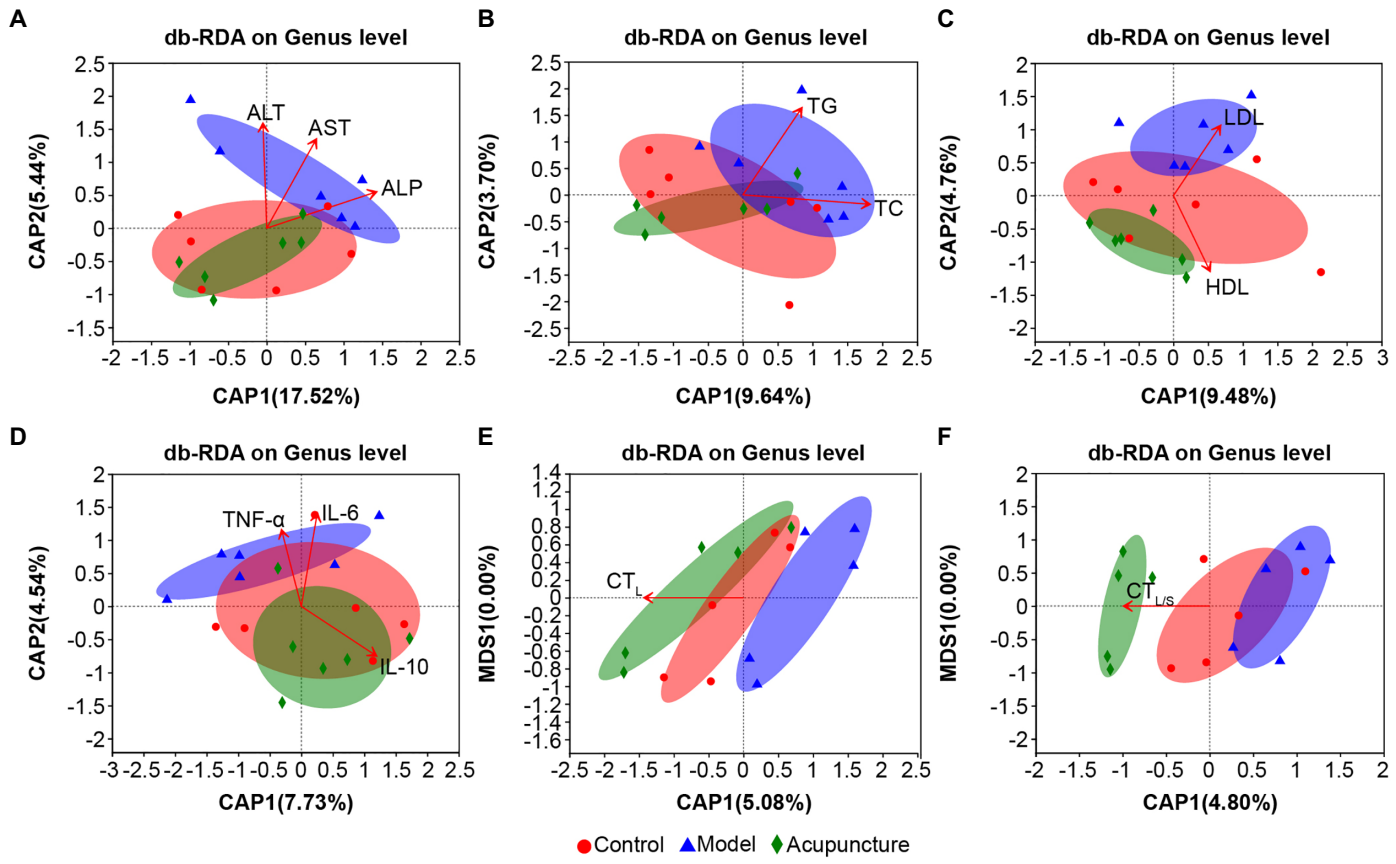
Distance-based redundancy analysis (db-RDA) on genus level of lipid metabolism parameters, inflammatory factors, and hepatic steatosis with intestinal microbiota. **(A)** Serum ALT, AST, and ALP. **(B)** Serum TC and TG. **(C)** Serum HDL and LDL. **(D)** Serum IL-6, TNF-α, and IL-10. **(E)** Liver CT (CT_L_) values. **(F)** CT values of liver to spleen ratio (CT_L/S_). The length of the red arrows represents the degree of the effect of serum lipid metabolism parameters, inflammation factors, and hepatic steatosis on the intestinal microbiota. The longer the arrow, the greater the correlation.

Overall, the bacterial community characteristics of the control and acupuncture groups were more similar than when each was compared with the model group (Figures 8A–F). The arrows derived *via* db-RDA for the correlation between the bacterial community and the lipid metabolism parameters ALT, AST (both *p* = 0.001, Figure 8A), and ALP (*p* = 0.002, Figure 8A); TC (*p* = 0.013, Figure 8B); TG (*p* = 0.001, Figure 8B); HDL (*p* = 0.001, Figure 8C); and LDL (*p* = 0.002, Figure 8C) presented significant distinctions. The inflammatory factors IL-6 (*p* = 0.001, Figure 8D), TNF-α (*p* = 0.005, Figure 8D), and IL-10 (*p* = 0.008, Figure 8D) were significantly correlated with the bacterial community. The hepatic steatosis markers CT_L_ (*p* = 0.001, Figure 8E) and CT_L/S_ (*p* = 0.002, Figure 8F) also presented close correlations with the bacterial community.

The above results indicate that lipid metabolism parameters, inflammatory factors, and hepatic steatosis are associated with the intestinal microbiota of NAFLD rats, yet the specific correlations need further elucidation.

#### 3.5.2. Spearman correlation analysis at the phylum and genus levels

To further demonstrate the relationship of specific species at the phylum and genus levels with lipid metabolism parameters, inflammatory factors, and hepatic steatosis, Spearman correlation analysis was performed using heatmaps.

**Figure 9:**
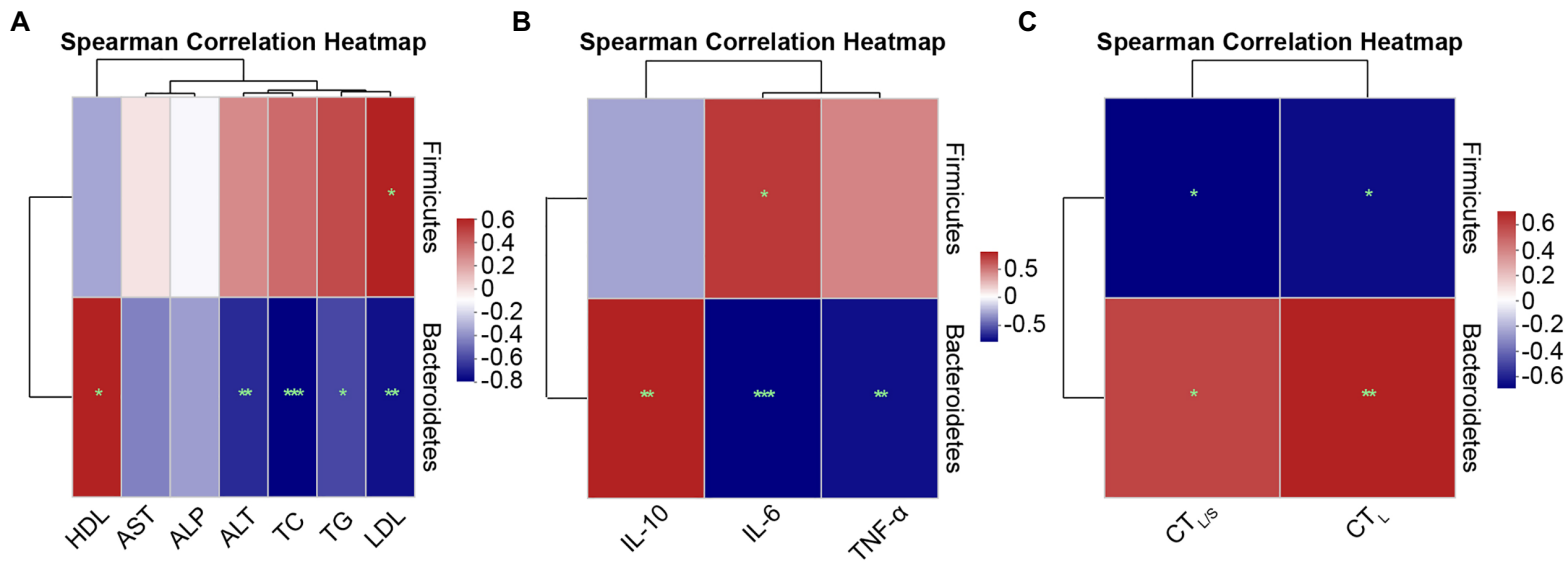
Spearman correlation heatmaps at the phylum level. **(A)** Correlation heatmap of the serum lipid metabolism parameters ALT, AST, ALP, TC, TG, HDL, and LDL with the intestinal microbiota. **(B)** Correlation heatmap of the inflammatory factors IL-6, TNF-α, and IL-10 with the intestinal microbiota. **(C)** Correlation heatmap of the hepatic steatosis indicators CT_L_ and CT_L/S_ with the intestinal microbiota. Red indicates a positive correlation, and blue indicates a negative correlation; the darker the color, the higher the correlation. Significance threshold, ^*^p < 0.05, ^**^p < 0.01, ^***^p < 0.001.

Spearman correlations at the phylum level are shown in Figure 9. Figure 9A shows the correlations between lipid metabolism parameters with Firmicutes and Bacteroidetes. Serum LDL (*r* = 0.52, *p* = 0.028) exhibited significant positive correlations with p-Firmicutes. The lipid metabolism parameters of ALT (*r* = −0.61, *p* = 0.007), TC (*r* = −0.72, *p* = 0.001), TG (*r* = −0.54, *p* = 0.020) and LDL (*r* = −0.67, *p* = 0.002) exhibited significant negative correlations with Bacteroidetes, and HDL (*r* = 0.52, *p* = 0.028) exhibited significant positive correlations with Bacteroidetes. Figure 9B shows the correlations between inflammatory factors and Firmicutes and Bacteroidetes. IL-6 (*r* = 0.59, *p* = 0.010) was positively correlated with Firmicutes. IL-6 (*r* = −0.74, *p* = 0.000) and TNF-α (*r* = −0.70, *p* = 0.001) were significantly negatively correlated with Bacteroidetes, while IL-10 (*r* = 0.66, *p* = 0.003) presented a significant positive correlation with Bacteroidetes. Figure 9C shows the correlations between hepatic steatosis and Firmicutes and Bacteroidetes. CT_L_ (*r* = −0.60, *p* = 0.018) and CT_L/S_ (*r* = −0.63, *p* = 0.012) exhibited significant negative correlations with Firmicutes, whereas CT_L_ (*r* = 0.68, *p* = 0.005) and CT_L/S_ (*r* = 0.58, *p* = 0.024) were significantly positively correlated with Bacteroidetes.

**Figure 10:**
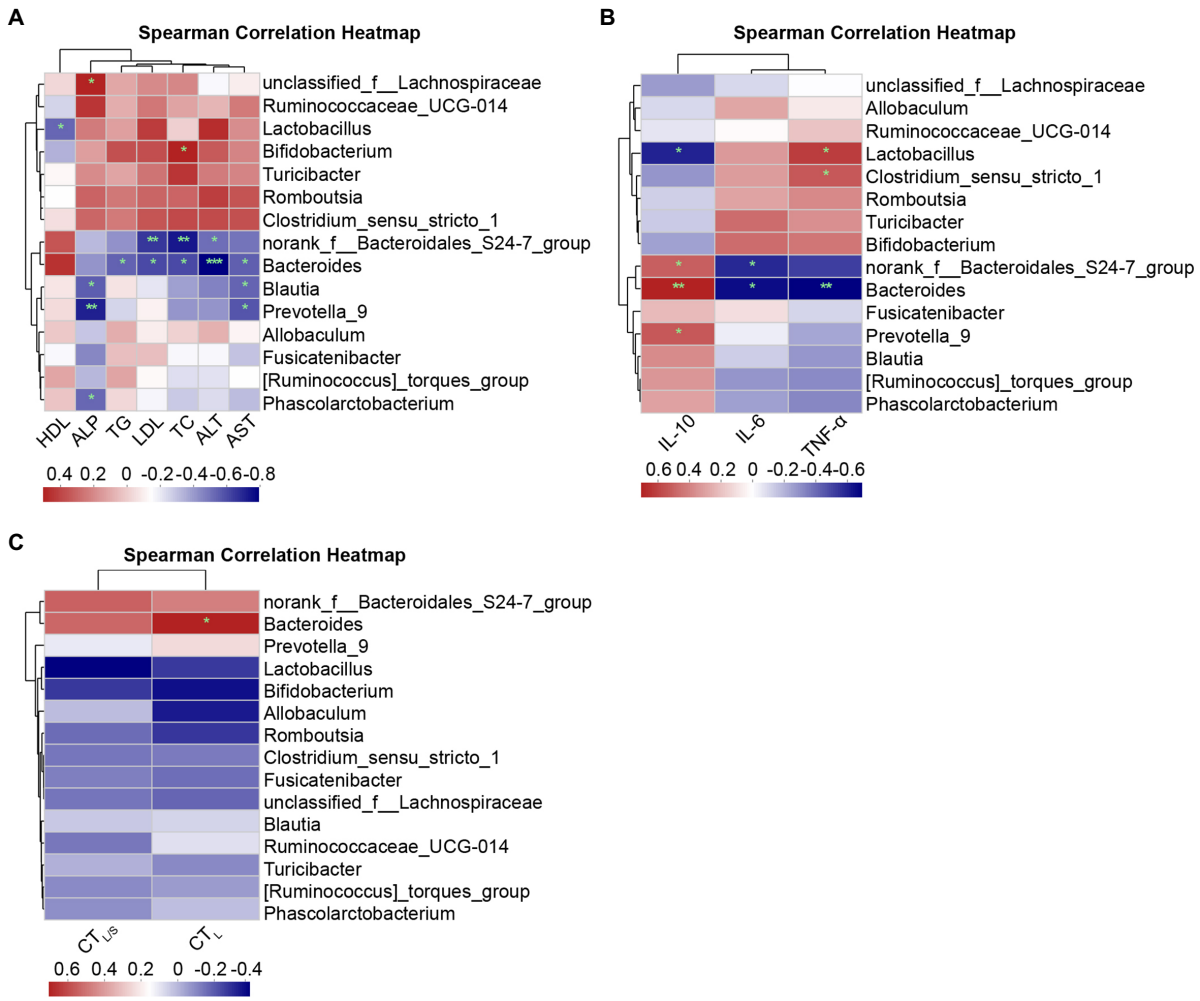
Spearman correlation heatmaps at the genus level. **(A)** Correlation heatmap of the serum lipid metabolism parameters ALT, AST, ALP, TC, TG, HDL, and LDL with the intestinal microbiota. **(B)** Correlation heatmap of the inflammatory factors IL-6, TNF-α, and IL-10 with the intestinal microbiota. **(C)** Correlation heatmap of the hepatic steatosis indicators CT_L_ and CT_L/S_ with the intestinal microbiota. Red indicates a positive correlation, and blue indicates a negative correlation; the darker the color, the higher the correlation. Significant threshold: ^*^p < 0.05, ^**^p < 0.01, ^***^p < 0.001.

Spearman correlations at the genus level are shown in Figure 10. Figure 10A shows the correlations between lipid metabolism parameters and the top abundant genera of the intestinal microbiota. ALP and unclassified_f_Lachnospiraceae (*r* = 0.49, *p* = 0.038), as well as TC and Bifidobacterium (*r* = 0.50, *p* = 0.037), presented significant positive correlations. Significant negative correlations were found for the following relationships: norank_f_Bacteroidales_S24-7_group with ALT (*r* = −0.47, *p =* 0.047), TC (*r* = −0.68, *p =* 0.002), and LDL (*r* = −0.61, *p =* 0.007); Bacteroides with ALT (*r* = −0.74, *p =* 0.000), AST (*r* = −0.51, *p =* 0.030), TC (*r* = −0.56, *p =* 0.015), LDL (*r* = −0.56, *p =* 0.015), and TG (*r* = −0.50, *p =* 0.033); Blautia and Prevotella_9 with AST (*r* = −0.50/−0.53, *p =* 0.035/0.022) and ALP (*r* = −0.51/−0.66, *p =* 0.030/0.003); and Phascolarctobacterium with ALP (*r* = −0.49, *p =* 0.038) exhibited a significant negative correlation. Figure 10B shows Spearman correlation between inflammatory factors and primary genera. Clostridium_sensu_stricto_1 (*r* = 0.49, *p =* 0.039) and Lactobacillus (*r* = 0.57, *p =* 0.014) were positively correlated to TNF-α, and Bacteroides (*r* = −0.61, *p =* 0.007) were negatively correlated to TNF-α. The genera norank_f_Bacteroidales_S24-7_group (*r* = 0.47, *p =* 0.048), Bacteroides (*r* = 0.64, *p =* 0.004), and Prevotella_9 (*r* = 0.49, *p =* 0.039) were positively correlated to IL-10, and Lactobacillus (*r* = −0.53, *p =* 0.024) exhibited negative correlations with IL-10; norank_f_Bacteroidales_S24-7_group (*r* = −0.51, *p =* 0.029) and Bacteroides (*r* = −0.58, *p =* 0.011) showed negative correlations with IL-6. Figure 10C shows the correlations between hepatic steatosis and genera. Bacteroides were positively correlated with CT_L_ (*r* = 0.61, *p* = 0.016).

The results above further support the notion that intestinal microbiota at the phylum and genus level is closely related to serum lipid metabolism parameters, inflammatory factors, and hepatic steatosis in NAFLD rats treated with acupuncture.

## 4. Discussion

We developed an HFD-induced NAFLD rat model to evaluate the effects of 6-week acupuncture treatment on fatty livers. Our results demonstrate that acupuncture effectively improved several indicators, such as serum lipids, liver function, and serum inflammatory factors. QCT was used to quantitatively evaluate liver fat content, while liver histopathology staining was performed to evaluate the lipidosis of liver tissues. Compared with serum markers and liver biopsy, the QCT method was selected as it is non-invasive, simple, and reliable (Marzuillo et al., 2015). The results show that acupuncture effectively improved hepatic steatosis and lipidosis, inhibited the inflammatory response, and slowed NAFLD progression.

Previous clinical and experimental studies (Jiang et al., 2011; Li et al., 2021b) have demonstrated that acupuncture has beneficial effects on lipid metabolism and type 2 diabetes complicated by NAFLD (Li et al., 2021a). The present study further proved that acupuncture could effectively improve lipid metabolism and alleviate hepatic steatosis in NAFLD rats, which is consistent with previously published findings (Zeng et al., 2014; Ma et al., 2020; Wang et al., 2022). However, we did not investigate the mechanism of action of acupuncture treatment in NAFLD, which should be explored in future studies.

The gastrointestinal tract interacts closely with the liver; the two are connected through the portal vein to form the intestine–liver axis. Therefore, disorders of intestinal microbiota and their related biology are seen as important regulatory factors in NAFLD pathophysiology (Tripathi et al., 2018). This study found that acupuncture could change the composition of the intestinal microbiota of NAFLD rats. The GB 26 acupoint was chosen in this study, owing to its anatomical position on the abdomen near the intestine, which may have contributed to its role in regulating intestinal microbiota.

Current studies on NAFLD and intestinal microbiota focus on the composition of microflora and their metabolic mechanisms (He et al., 2021), and few studies have explored acupuncture’s role in regulating intestinal microbiota. A study by Xie et al. found that acupuncture could improve intestinal microbiota diversity (Xie et al., 2020). Another clinical study on intestinal microbiota and persistent NAFLD found declining microorganism diversity in NAFLD patients (Kim et al., 2019). We found no significant differences in microbiota diversity among the model, control, and acupuncture groups. This may be explained by the fact that differences among individual microorganisms are greater than those among communities, or our findings may have been influenced by sample size. Current studies on community diversity are inconsistent, indicating the need for further research on microbiota species diversity relating to NAFLD using larger sample sizes.

In the human intestinal microbiome, Firmicutes and Bacteroidetes are dominant, comprising over 90% of the total community (Eckburg et al., 2005). Therefore, the F/B ratio often serves as a marker of intestinal microbiome performance (Hildebrandt et al., 2009). Clinical studies found that the F/B ratio of NAFLD patients was positively correlated to steatosis (Jasirwan et al., 2021). The F/B ratio increased in HFD-induced NAFLD mice and decreased following bilberry anthocyanin intervention (Nakano et al., 2020), supporting the trend observed in this study. Furthermore, although acupuncture did not significantly influence the relative abundance of Firmicutes, it significantly increased the abundance of probiotic Bacteroidetes, demonstrating an effective improvement in NAFLD intestinal microbiota by acupuncture.

Bacteroidales_S24-7_group bacteria can produce butyrate, which may slow NAFLD progression (Endo et al., 2013; Zhou et al., 2017). Our study found that the top three dominant microbiota families among the control, model, and acupuncture groups were Lachnospiraceae, Erysipelotrichaceae, and Peptostreptococcaceae, without significant differences; however, acupuncture significantly increased the abundance of Bacteroidales_S24-7_group, Prevotellaceae, and Bacteroidaceae, indicating an improved probiotics ratio in NAFLD rats after treatment.

The genus Blautia is considered a probiotic that may improve metabolic disorders as it produces butyrate, which benefits intestinal health (Liu et al., 2021). A study from Japan found that increased Blautia abundance had a significant negative correlation with visceral fat accumulation (Ozato et al., 2019)*. HFD-fed mice administered fermented celery (Apium graveolens L.) juice intervention exhibited a significant increase in Ruminococcaceae_UCG-014 abundance* (Zhao et al., 2021). *Bacteroides may potentially treat metabolic disorders such as diabetes and obesity* (Yang et al., 2017). Glucose metabolism improvement induced by dietary fibers may be related to Prevotella abundance increase, yet some studies also link Prevotella to adverse physiological effects such as insulin resistance (Pedersen et al., 2016). The current study showed that, at the genus level, acupuncture could significantly improve the relative abundance of the probiotics Blautia, Prevotella_9, norank_f_Bacteroidales_S24-7_group, and Bacteroides and decrease the abundance of Ruminococcaceae_UCG-014; therefore, we hypothesized that acupuncture could improve hepatic steatosis and lipidosis, as well as inhibit the inflammatory response by regulating composition of some intestinal microbiota, so as to slow NAFLD progression.

As revealed by the db-RDA, serum lipid metabolism parameters, hepatic steatosis, and inflammatory factors were closely correlated to the bacterial community. The microbiota community distances among the three groups were distinct, and species in the acupuncture group were more similar to those in the control group than those in the model group. This further demonstrated that acupuncture potentially regulates lipid metabolism, inflammatory responses, and hepatic steatosis by influencing intestinal microbiota. However, this correlation analysis is only from the general level, and more specific correlations should be performed.

Using Spearman correlation, we found that lipid metabolism and inflammation were mostly related to changes in the phylum Bacteroidetes, while hepatic steatosis was related to both Firmicutes and Bacteroidetes. At the genus level, lipid metabolism and inflammation were mostly correlated with norank_f_Bacteroidales_S24-7_group and Bacteroides, as well as Lactobacillus, Clostridium_sensu_stricto_1g_Blautia, and Prevotella_9. Hepatic steatosis was mostly correlated with Bacteroides. These correlations indicate that NAFLD markers that were changed by acupuncture are closely related to the intestinal microbiota. However, fecal bacteria transplantation experiments are needed to clarify the influence of changed intestinal microbiota on NAFLD. Correlations with each marker vary across genera, and each species within each genus exerts different functions in the intestine (Walker et al., 2014).

There are two main limitations of this study. First, present studies on acupuncture in treating NAFLD are still insufficient, with a critical lack of data on relevant genera and correlations between NAFLD-related markers and intestinal microbiota. Additionally, larger clinical and experimental studies are required to verify the effectiveness of acupuncture and to investigate its potential mechanisms. Therefore, more advanced sequencing methods, such as metagenomics, are required to further analyze the influence of acupuncture on intestinal microbiota in NAFLD and clarify the roles of specific genera.

## 5. Conclusion

Acupuncture can improve hepatic steatosis in HFD-induced NAFLD rats. We demonstrated that acupuncture might have a beneficial effect on NAFLD by improving intestinal microbiota. Our findings also support the validity of selecting acupoint GB 26 in this study. Further studies are recommended to validate the regulatory role of acupuncture on the intestinal microbiota of NAFLD rats by fecal bacteria transplantation and investigate the role of intestinal microbiota metabolites.

## Data availability statement

The datasets presented in this study can be found in online repositories. The names of the repository/repositories and accession number(s) can be found in the article/supplementary material.

## Ethics statement

The animal study was reviewed and approved by Research Animal Care Committee of the Institute of Chinese People’s Liberation Army Center of Disease Control and Prevention.

## Author contributions

HF and HH designed and guided the experiment study, they contributed equally to this work and shared the corresponding authors. HW carried out the experiment procedures, analyzed the data, and completed the manuscript. QW guided the experimental modeling, manuscript writing, and assisted in statistical analysis. CL and LP carried out the experimental modeling and acupuncture intervention, and all the authors participated in manuscript revisions. All authors contributed to the article and approved the submitted version.

## Funding

This study was supported by grants from the Beijing Administration of Traditional Chinese Medicine, Chinese Medicine “3 + 3” Inheritance Project “JiasanYang Famous Research Studio,” Beijing Municipal Administration of Hospitals Incubating Program (PX2023019) and Beijing Natural Science Foundation (7232047).

## Conflict of interest

QW was employed by Chinese People’s Liberation Army Center of Disease Control and Prevention.

The remaining authors declare that the research was conducted in the absence of any commercial or financial relationships that could be construed as a potential conflict of interest.

The reviewer CL declared a shared parent affiliation with the author HF to the handling editor at the time of review.

## Publisher’s note

All claims expressed in this article are solely those of the authors and do not necessarily represent those of their affiliated organizations, or those of the publisher, the editors and the reviewers. Any product that may be evaluated in this article, or claim that may be made by its manufacturer, is not guaranteed or endorsed by the publisher.

## References

[ref1] Asrani S. K. Devarbhavi H. Eaton J. Kamath P. S. (2019). Burden of liver diseases in the world. J. Hepatol. 70, 151–171. doi: 10.1016/j.jhep.2018.09.014 30266282

[ref2] Ballestri S. Nascimbeni F. Romagnoli D. Lonardo A. (2016). The independent predictors of non-alcoholic steatohepatitis and its individual histological features: insulin resistance, serum uric acid, metabolic syndrome, alanine aminotransferase and serum total cholesterol are a clue to pathogenesis and candidate targets for treatment. Hepatol. Res. 46, 1074–1087. doi: 10.1111/hepr.12656, PMID: 26785389

[ref3] Chen P. Zhong X. Dai Y. Tan M. Zhang G. Ke X.. (2021). The efficacy and safety of acupuncture in non-alcoholic fatty liver disease: a systematic review and meta-analysis of randomized controlled trials. Medicine (Baltimore) 100:e27050. doi: 10.1097/md.0000000000027050, PMID: 34559098 PMC8462626

[ref4] Dong C. Zhang C. R. Xue B. Y. Miu W. F. Fang N. Y. Li K.. (2020). Electroacupuncture combined with lifestyle control on obese nonalcoholic fatty liver disease: a randomized controlled trial. Zhongguo Zhen Jiu 40, 129–134. doi: 10.13703/j.0255-2930.20190201-k00034, PMID: 32100496

[ref5] Eckburg P. B. Bik E. M. Bernstein C. N. Purdom E. Dethlefsen L. Sargent M.. (2005). Diversity of the human intestinal microbial flora. Science 308, 1635–1638. doi: 10.1126/science.1110591, PMID: 15831718 PMC1395357

[ref6] Endo H. Niioka M. Kobayashi N. Tanaka M. Watanabe T. (2013). Butyrate-producing probiotics reduce nonalcoholic fatty liver disease progression in rats: new insight into the probiotics for the gut-liver axis. PLoS One 8:e63388. doi: 10.1371/journal.pone.0063388, PMID: 23696823 PMC3656030

[ref7] Estes C. Razavi H. Loomba R. Younossi Z. Sanyal A. J. (2018). Modeling the epidemic of nonalcoholic fatty liver disease demonstrates an exponential increase in burden of disease. Hepatology 67, 123–133. doi: 10.1002/hep.29466, PMID: 28802062 PMC5767767

[ref8] Han J. Guo X. Meng X. J. Zhang J. Yamaguchi R. Motoo Y.. (2020). Acupuncture improved lipid metabolism by regulating intestinal absorption in mice. World J. Gastroenterol. 26, 5118–5129. doi: 10.3748/wjg.v26.i34.5118, PMID: 32982113 PMC7495030

[ref9] He L. H. Yao D. H. Wang L. Y. Zhang L. Bai X. L. (2021). Gut microbiome-mediated alteration of immunity, inflammation, and metabolism involved in the regulation of non-alcoholic fatty liver disease. Front. Microbiol. 12:761836. doi: 10.3389/fmicb.2021.761836, PMID: 34795655 PMC8593644

[ref10] Hildebrandt M. A. Hoffmann C. Sherrill-Mix S. A. Keilbaugh S. A. Hamady M. Chen Y. Y.. (2009). High-fat diet determines the composition of the murine gut microbiome independently of obesity. Gastroenterology 137, 1716–1724.e1711-1712. doi: 10.1053/j.gastro.2009.08.042, PMID: 19706296 PMC2770164

[ref11] Hoyles L. Fernández-Real J. M. Federici M. Serino M. Abbott J. Charpentier J.. (2018). Molecular phenomics and metagenomics of hepatic steatosis in non-diabetic obese women. Nat. Med. 24, 1070–1080. doi: 10.1038/s41591-018-0061-3, PMID: 29942096 PMC6140997

[ref12] Jasirwan C. O. M. Muradi A. Hasan I. Simadibrata M. Rinaldi I. (2021). Correlation of gut Firmicutes/Bacteroidetes ratio with fibrosis and steatosis stratified by body mass index in patients with non-alcoholic fatty liver disease. Biosci. Microbiota Food Health 40, 50–58. doi: 10.12938/bmfh.2020-046, PMID: 33520569 PMC7817510

[ref13] Jiang Y. L. Ning Y. Liu Y. Y. Wang Y. Zhang Z. Yin L. M.. (2011). Effects of preventive acupuncture on streptozotocin-induced hyperglycemia in rats. J. Endocrinol. Investig. 34, e355–e361. doi: 10.3275/7859, PMID: 21750401

[ref14] Kim H. N. Joo E. J. Cheong H. S. Kim Y. Kim H. L. Shin H.. (2019). Gut microbiota and risk of persistent nonalcoholic fatty liver diseases. J. Clin. Med. 8:1089. doi: 10.3390/jcm8081089, PMID: 31344854 PMC6722749

[ref15] Le Roy T. Llopis M. Lepage P. Bruneau A. Rabot S. Bevilacqua C.. (2013). Intestinal microbiota determines development of non-alcoholic fatty liver disease in mice. Gut 62, 1787–1794. doi: 10.1136/gutjnl-2012-303816 23197411

[ref16] Lee S. S. Park S. H. (2014). Radiologic evaluation of nonalcoholic fatty liver disease. World J. Gastroenterol. 20, 7392–7402. doi: 10.3748/wjg.v20.i23.7392, PMID: 24966609 PMC4064084

[ref17] Li X. Jia H. X. Yin D. Q. Zhang Z. J. (2021b). Acupuncture for metabolic syndrome: systematic review and meta-analysis. Acupunct. Med. 39, 253–263. doi: 10.1177/0964528420960485, PMID: 33032446

[ref18] Li M. Yao L. Huang H. Wang G. Yu B. Zheng H.. (2021a). Acupuncture for type 2 diabetes mellitus with nonalcoholic fatty liver disease: a protocol for systematic review and meta-analysis. Medicine (Baltimore) 100:e26043. doi: 10.1097/md.0000000000026043, PMID: 34032730 PMC8154503

[ref19] Liu X. Mao B. Gu J. Wu J. Cui S. Wang G.. (2021). Blautia-a new functional genus with potential probiotic properties? Gut Microbes 13, 1–21. doi: 10.1080/19490976.2021.1875796, PMID: 33525961 PMC7872077

[ref20] Ma B. Li P. An H. Song Z. (2020). Electroacupuncture attenuates liver inflammation in nonalcoholic fatty liver disease rats. Inflammation 43, 2372–2378. doi: 10.1007/s10753-020-01306-w, PMID: 32737656

[ref21] Magoč T. Salzberg S. L. (2011). FLASH: fast length adjustment of short reads to improve genome assemblies. Bioinformatics 27, 2957–2963. doi: 10.1093/bioinformatics/btr507, PMID: 21903629 PMC3198573

[ref22] Marzuillo P. Grandone A. Perrone L. Miraglia Del Giudice E. (2015). Controversy in the diagnosis of pediatric non-alcoholic fatty liver disease. World J. Gastroenterol. 21, 6444–6450. doi: 10.3748/wjg.v21.i21.6444, PMID: 26074683 PMC4458755

[ref23] Nakano H. Wu S. Sakao K. Hara T. He J. Garcia S.. (2020). Bilberry anthocyanins ameliorate NAFLD by improving dyslipidemia and gut microbiome dysbiosis. Nutrients 12:3252. doi: 10.3390/nu12113252, PMID: 33114130 PMC7690841

[ref24] Noureddin M. Vipani A. Bresee C. Todo T. Kim I. K. Alkhouri N.. (2018). NASH leading cause of liver transplant in women: updated analysis of indications for liver transplant and ethnic and gender variances. Am. J. Gastroenterol. 113, 1649–1659. doi: 10.1038/s41395-018-0088-6, PMID: 29880964 PMC9083888

[ref25] Ozato N. Saito S. Yamaguchi T. Katashima M. Tokuda I. Sawada K.. (2019). Blautia genus associated with visceral fat accumulation in adults 20-76 years of age. NPJ Biofilms Microbiomes 5:28. doi: 10.1038/s41522-019-0101-x, PMID: 31602309 PMC6778088

[ref26] Paik J. M. Golabi P. Younossi Y. Mishra A. Younossi Z. M. (2020). Changes in the global burden of chronic liver diseases from 2012 to 2017: the growing impact of NAFLD. Hepatology 72, 1605–1616. doi: 10.1002/hep.31173, PMID: 32043613

[ref27] Pedersen H. K. Gudmundsdottir V. Nielsen H. B. Hyotylainen T. Nielsen T. Jensen B. A.. (2016). Human gut microbes impact host serum metabolome and insulin sensitivity. Nature 535, 376–381. doi: 10.1038/nature18646, PMID: 27409811

[ref28] Sharpton S. R. Ajmera V. Loomba R. (2019). Emerging role of the gut microbiome in nonalcoholic fatty liver disease: from composition to function. Clin. Gastroenterol. Hepatol. 17, 296–306. doi: 10.1016/j.cgh.2018.08.065, PMID: 30196156 PMC6314895

[ref29] Simon T. G. Roelstraete B. Khalili H. Hagström H. Ludvigsson J. F. (2021). Mortality in biopsy-confirmed nonalcoholic fatty liver disease: results from a nationwide cohort. Gut 70, 1375–1382. doi: 10.1136/gutjnl-2020-322786, PMID: 33037056 PMC8185553

[ref30] Tripathi A. Debelius J. Brenner D. A. Karin M. Loomba R. Schnabl B.. (2018). The gut-liver axis and the intersection with the microbiome. Nat. Rev. Gastroenterol. Hepatol. 15, 397–411. doi: 10.1038/s41575-018-0011-z, PMID: 29748586 PMC6319369

[ref31] Walker A. W. Duncan S. H. Louis P. Flint H. J. (2014). Phylogeny, culturing, and metagenomics of the human gut microbiota. Trends Microbiol. 22, 267–274. doi: 10.1016/j.tim.2014.03.001 24698744

[ref32] Wang G. Li M. Yu S. Guan M. Ma S. Zhong Z.. (2022). Tandem mass tag-based proteomics analysis of type 2 diabetes mellitus with non-alcoholic fatty liver disease in mice treated with acupuncture. Biosci. Rep. 42:BSR20212248. doi: 10.1042/bsr20212248, PMID: 34981123 PMC8762347

[ref33] Wong R. J. Cheung R. Ahmed A. (2014). Nonalcoholic steatohepatitis is the most rapidly growing indication for liver transplantation in patients with hepatocellular carcinoma in the U.S. Hepatology 59, 2188–2195. doi: 10.1002/hep.26986, PMID: 24375711

[ref34] Xie L. L. Zhao Y. L. Yang J. Cheng H. Zhong Z. D. Liu Y. R.. (2020). Electroacupuncture prevents osteoarthritis of high-fat diet-induced obese rats. Biomed. Res. Int. 2020:9380965. doi: 10.1155/2020/9380965, PMID: 32724821 PMC7366230

[ref35] Yang J. Y. Lee Y. S. Kim Y. Lee S. H. Ryu S. Fukuda S.. (2017). Gut commensal Bacteroides acidifaciens prevents obesity and improves insulin sensitivity in mice. Mucosal Immunol. 10, 104–116. doi: 10.1038/mi.2016.42, PMID: 27118489

[ref36] Younossi Z. M. Golabi P. de Avila L. Paik J. M. Srishord M. Fukui N.. (2019). The global epidemiology of NAFLD and NASH in patients with type 2 diabetes: a systematic review and meta-analysis. J. Hepatol. 71, 793–801. doi: 10.1016/j.jhep.2019.06.021, PMID: 31279902

[ref37] Yu M. Li G. Tang C. L. Gao R. Q. Feng Q. T. Cao J. (2017). Effect of Electroacupunctrue stimulation at Fenglong (ST 40) on expression of SREBP-1 c in non-alcoholic fatty liver disease rats. Zhen Ci Yan Jiu 42, 308–314. PMID: 29072011

[ref38] Yu J. S. Youn G. S. Choi J. Kim C. H. Kim B. Y. Yang S. J.. (2021). Lactobacillus lactis and Pediococcus pentosaceus-driven reprogramming of gut microbiome and metabolome ameliorates the progression of non-alcoholic fatty liver disease. Clin. Transl. Med. 11:e634. doi: 10.1002/ctm2.634, PMID: 34965016 PMC8715831

[ref39] Zeng M. D. Fan J. G. Lu L. G. Li Y. M. Chen C. W. Wang B. Y.. (2008). Guidelines for the diagnosis and treatment of nonalcoholic fatty liver diseases. J. Dig. Dis. 9, 108–112. doi: 10.1111/j.1751-2980.2008.00331.x 18419645

[ref40] Zeng Z. H. Zeng M. H. Huang X. K. Chen R. Yu H. (2014). Effect of electroacupuncture stimulation of back-shu points on expression of TNF-alpha and lipid peroxidation reaction in the liver tissue in non-alcoholic fatty liver disease rats. Zhen Ci Yan Jiu 39, 288–292. PMID: 25219124

[ref41] Zhang S. Y. Li L. L. Hu X. Tang H. T. (2020). Effect of acupuncture on oxidative stress and apoptosis-related proteins in obese mice induced by high-fat diet. Zhongguo Zhen Jiu 40, 983–988. doi: 10.13703/j.0255-2930.20190821-0006, PMID: 32959595

[ref42] Zhao D. Cao J. Jin H. Shan Y. Fang J. Liu F. (2021). Beneficial impacts of fermented celery (Apium graveolens L.) juice on obesity prevention and gut microbiota modulation in high-fat diet fed mice. Food Funct. 12, 9151–9164. doi: 10.1039/d1fo00560j, PMID: 34606532

[ref43] Zhou D. Pan Q. Xin F. Z. Zhang R. N. He C. X. Chen G. Y.. (2017). Sodium butyrate attenuates high-fat diet-induced steatohepatitis in mice by improving gut microbiota and gastrointestinal barrier. World J. Gastroenterol. 23, 60–75. doi: 10.3748/wjg.v23.i1.60, PMID: 28104981 PMC5221287

